# *catena*-Poly[[aqua­lithium(I)]-μ-3-carb­oxy-5,6-di­methyl­pyrazine-2-carboxyl­ato-κ^4^ *O* ^2^,*N* ^1^:*O* ^3^,*N* ^4^]

**DOI:** 10.1107/S1600536813030493

**Published:** 2013-11-13

**Authors:** Wojciech Starosta, Janusz Leciejewicz

**Affiliations:** a Institute of Nuclear Chemistry and Technology, ul. Dorodna 16, 03-195 Warszawa, Poland

## Abstract

The asymmetric unit of the title compound, [Li(C_8_H_6_N_2_O_4_)(H_2_O)]_*n*_, comprises three Li cations, two of which are located on a twofold rotation axis, two carboxylate anions and three water mol­ecules, of which two are situated on the twofold rotation axis being aqua ligands. Both carboxylate anions are in μ_2_-bridging mode. All Li ions show a trigonal–bipyramidal coordination mode; the two located in special positions are bridged through *N*,*O*-bonding sites generating a polymeric ribbon along the *c-*axis direction. The Li cation in a general position creates an independent polymeric ribbon through *N*,*O*-bonding sites of the two symmetry-related ligands; the trigonal–bipyramidal coordination is completed by an aqua ligand. In both carboxylate anions, a carboxyl­ate and a carb­oxy­lic acid group form an intra­molecular hydrogen bond. The polymeric ribbons running along [001] are inter­connected by hydrogen bonds in which the water mol­ecules act as donors and carboxyl­ate O atoms act as acceptors, giving rise to a three-dimensional architecture.

## Related literature

For the structures of lithium complexes with pyrazine-2,3-di­carboxyl­ate ligands, see: Tombul *et al.* (2008 ▶); Tombul & Güven (2009) ▶; Starosta & Leciejewicz (2011 ▶, 2013 ▶). The structure of 5,6-di­methyl­pyrazine-2,3-di­carb­oxy­lic acid dihydrate was reported by Vishwershwar *et al.* (2001 ▶). 
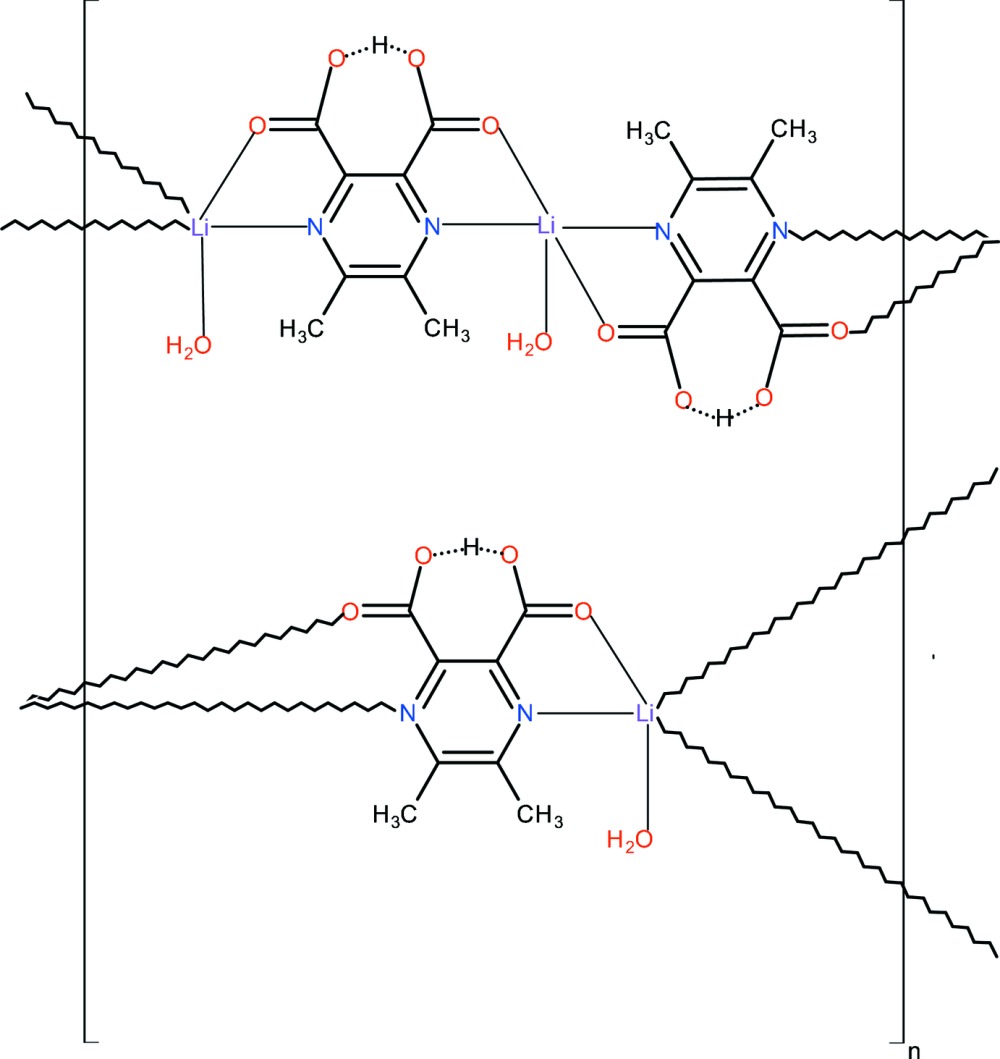


## Experimental

### 

#### Crystal data

[Li(C_8_H_7_N_2_O_4_)(H_2_O)]
*M* *_r_* = 220.11
Monoclinic, 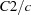

*a* = 16.9052 (2) Å
*b* = 16.7980 (2) Å
*c* = 14.3805 (2) Å
β = 97.272 (1)°
*V* = 4050.83 (9) Å^3^
*Z* = 16
Cu *K*α radiation
μ = 1.02 mm^−1^
*T* = 293 K
0.24 × 0.07 × 0.02 mm

#### Data collection

Oxford Diffraction Xcalibur Ruby diffractometer
Absorption correction: analytical [*CrysAlis PRO* (Oxford Diffraction, 2010 ▶), using a multifaceted crystal model (Clark & Reid, 1995 ▶)] *T*_min_ = 0.738, *T*_max_ = 0.958
37580 measured reflections
3808 independent reflections
2898 reflections with *I* > 2σ(*I*)
*R*_int_ = 0.039

#### Refinement

*R*[*F* ^2^ > 2σ(*F* ^2^)] = 0.040
*wR*(*F* ^2^) = 0.110
*S* = 0.97
3808 reflections
319 parameters
H atoms treated by a mixture of independent and constrained refinement
Δρ_max_ = 0.18 e Å^−3^
Δρ_min_ = −0.19 e Å^−3^

### 

Data collection: *CrysAlis PRO* (Oxford Diffraction, 2010 ▶); cell refinement: *CrysAlis PRO*; data reduction: *CrysAlis PRO*; program(s) used to solve structure: *SHELXS97* (Sheldrick, 2008 ▶); program(s) used to refine structure: *SHELXL97* (Sheldrick, 2008 ▶); molecular graphics: *SHELXTL* (Sheldrick, 2008 ▶); software used to prepare material for publication: *SHELXTL*.

## Supplementary Material

Crystal structure: contains datablock(s) I, New_Global_Publ_Block. DOI: 10.1107/S1600536813030493/kp2460sup1.cif

Structure factors: contains datablock(s) I. DOI: 10.1107/S1600536813030493/kp2460Isup2.hkl

Additional supplementary materials:  crystallographic information; 3D view; checkCIF report

## Figures and Tables

**Table 1:** Selected bond lengths (Å)

Li11—O15	1.915 (4)
Li11—O11	1.9473 (19)
Li11—O11^i^	1.9472 (19)
Li11—N11^i^	2.3128 (14)
Li11—N11	2.3129 (14)
Li12—O16	1.884 (4)
Li12—O13	1.920 (2)
Li12—O13^ii^	1.920 (2)
Li12—N14^ii^	2.3446 (12)
Li12—N14	2.3446 (12)
Li21—O25	1.902 (3)
Li21—O21	1.923 (3)
Li21—O24^iii^	1.949 (3)
Li21—N21	2.276 (3)
Li21—N24^iii^	2.324 (3)

Symmetry codes: (i) 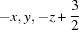; (ii) 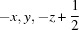; (iii) 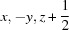.

**Table 2:** Hydrogen-bond geometry (Å, °)

*D*—H⋯*A*	*D*—H	H⋯*A*	*D*⋯*A*	*D*—H⋯*A*
O22—H221⋯O23	1.18 (3)	1.19 (3)	2.3693 (18)	177 (3)
O12—H121⋯O14	1.14 (3)	1.24 (3)	2.3777 (18)	177 (3)
O16—H161⋯O22^iv^	0.82 (3)	2.01 (3)	2.8268 (18)	173 (3)
O15—H151⋯O24^iv^	0.85 (3)	2.22 (4)	2.989 (2)	150 (3)
O15—H151⋯O23^iv^	0.85 (3)	2.34 (3)	3.1201 (14)	153 (3)
O25—H251⋯O12^v^	0.91 (3)	2.03 (3)	2.938 (2)	174 (2)
O25—H252⋯O14^ii^	0.80 (3)	2.20 (3)	2.975 (2)	163 (3)
O25—H252⋯O13^ii^	0.80 (3)	2.43 (3)	3.078 (2)	139 (3)

Symmetry codes: (ii) 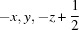; (iv) 
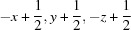
; (v) 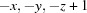.

**Fig. 1.**
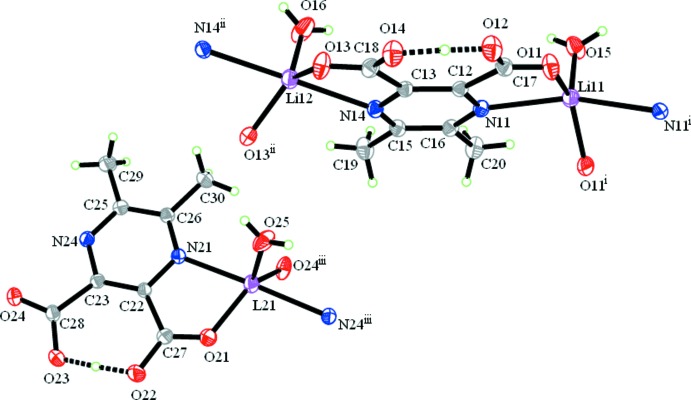
Structural units of the title complex with atom labelling scheme and 50% probability displacement ellipsoids. Symmetry code: (i) -x, y, -z + 3/2; (ii) -x, y, -z + 1/2; (iii): x, -y, z + 1/2.

**Fig. 2.**
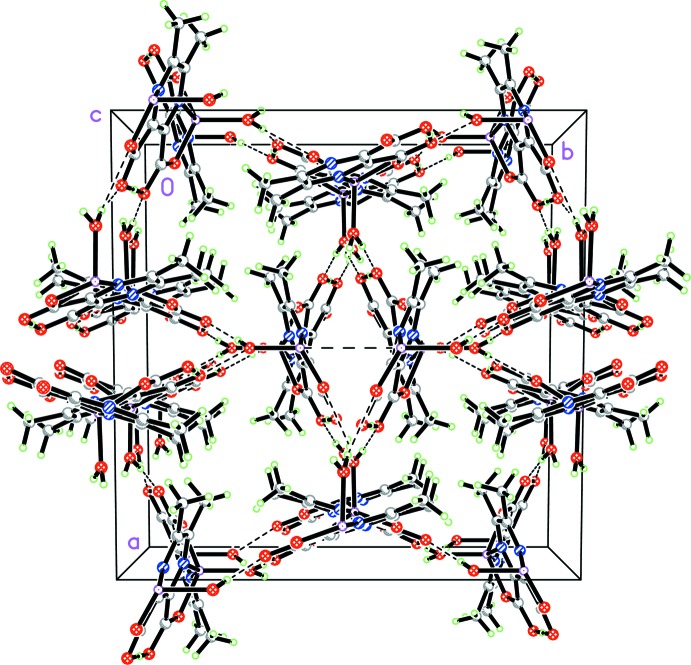
The packing of molecular ribbons viewed along the [001] direction.

## supplementary crystallographic information

### 1. Comment

The asymmetric unit of the title compound contains three Li cations (two of them located on the rotation twofold axes), two ligand and three water molecules (two of them located on the rotation twofold axes). Two water-coordinated Li ions bridged by a ligand *via* both N,*O* bonding sites form the type 1 molecular ribbon; the type 2 ribbon is built of units composed of a water-coordinated Li cation and a ligand which also uses its N,*O* bonding sites (Fig.1, Table 1). Both ligands act in µ_2_ bridging mode. All three Li cations show slightly distorted trigonal bipyramidal coordination geometry. The Li11 cation is situated in the equatorial plane composed of O11, O11^ii^ and O15 atoms; N11 and N11^ii^ atoms are in the apical positions. The equatorial plane of Li12 coordination is formed by O13, O13^iii^ and O16 atoms and N14and N14^iii^ atoms are at the apices; Li12 is coplanar with the equatorial ligand plane. However, Li21 cation is 0.0142 (2) Å out of the equatorial plane of O21, O21^i^ and O25 atoms; N21 and N21^i^ are at the apices. The Li—O and Li—N bond distances (Table 2), fall in the range observed in the structures of other Li complexes with diazine carboxylate ligands. Methyl carbon and pyrazine ring atoms of both ligands are coplanar with r.m.s of 0.0062 (1) Å in the ligand 1 and 0.0193 (2) Å in the ligand 2. The carboxylic groups C11/O11/O12 and C18/O13/O14 form with the ligand 1 ring dihedral angles of 6.1 (1)° and 10.9 (1)°, respectively. The dihedral angles between ligand 2 ring and carboxyl groups C27/O21/O22 and C28/O23/O24 are 1.2 (1)° and 9.0 (1)°, respectively. In both ligands the second carboxyl O atoms remain protonated and act as donors in the short intramolecular hydrogen bonds. Bond distances and bond angles within the ligand molecules do not differ from those reported in the structure of the parent acid (Vishwershwar *et al.*, 2001). Two ribbons of the same type form pairs which propagate in the [001] direction. The planes of ribbon 1 and ribbon 2 pairs are inclined 91.9 (3)° each to the other (Fig. 2). They are held together by a system of hydrogen bonds in which water molecules act as donors and carboxyl O atoms as acceptors (Table 3).

Molecular ribbons built of Li ions bridged by pyrazine-2,3-dicarboxylate ligand *via* its both N,*O* bonding sites constitute the building units of four catenated polymeric structures of Li complexes with this ligand. In all of them, Li coordination is trigonal bipyramidal (Tombul *et al.*, 2008), (Tombul & Güven, 2009), (Starosta & Leciejewicz, 2011), (Starosta & Leciejewicz, 2013).

### 2. Experimental

To 50 mL of a solution of 5,6-dimethylpyrazine-2,3-dicarboxylic acid dihydrate in doubly distilled water an 1 N solution of LiOH was added by drops until pH reached 5.5. Then, the solution was boiled under reflux with stirring for 5 h. After cooling to room temperature, the solution was left to crystallize. The material which was found after evaporation to dryness was recrystallized from cold water. The obtained single-crystal blocks were washed with cold ethanol and dried in air.

### 3. Refinement

Hydrogen atoms belonging to water molecules and the carboxylic group were located in a difference map and refined isotropically while twelve methyl H atoms were located at calculated positions and treated as riding on the parent C atoms with C—H=0.96 Å.

### Crystal data

**Table:**

[Li(C_8_H_7_N_2_O_4_)(H_2_O)]	*Z* = 16
*M**_r_* = 220.11	*F*(000) = 1824
Monoclinic, *C*2/*c*	*D*_x_ = 1.444 Mg m^−^^3^
Hall symbol: -C 2yc	Cu *K*α radiation, λ = 1.54178 Å
*a* = 16.9052 (2) Å	µ = 1.02 mm^−^^1^
*b* = 16.7980 (2) Å	*T* = 293 K
*c* = 14.3805 (2) Å	Plate, colourless
β = 97.272 (1)°	0.24 × 0.07 × 0.02 mm
*V* = 4050.83 (9) Å^3^	

### Data collection

**Table:**

Oxford Diffraction Xcalibur Ruby diffractometer	3808 independent reflections
Radiation source: Enhance (Cu) X-ray Source	2898 reflections with *I* > 2σ(*I*)
Graphite monochromator	*R*_int_ = 0.039
Detector resolution: 10.4922 pixels mm^-1^	θ_max_ = 70.1°, θ_min_ = 3.7°
rotation method, ω scans	*h* = −20→20
Absorption correction: analytical [*CrysAlis PRO* (Oxford Diffraction, 2010), using a multifaceted crystal model (Clark & Reid, 1995)]	*k* = −20→20
*T*_min_ = 0.738, *T*_max_ = 0.958	*l* = −15→17
37580 measured reflections	

### Refinement

**Table:**

Refinement on *F*^2^	Primary atom site location: structure-invariant direct methods
Least-squares matrix: full	Secondary atom site location: difference Fourier map
*R*[*F*^2^ > 2σ(*F*^2^)] = 0.040	Hydrogen site location: inferred from neighbouring sites
*wR*(*F*^2^) = 0.110	H atoms treated by a mixture of independent and constrained refinement
*S* = 0.97	*w* = 1/[σ^2^(*F*_o_^2^) + (0.0785*P*)^2^] where *P* = (*F*_o_^2^ + 2*F*_c_^2^)/3
3808 reflections	(Δ/σ)_max_ < 0.001
319 parameters	Δρ_max_ = 0.18 e Å^−^^3^
0 restraints	Δρ_min_ = −0.19 e Å^−^^3^

### Special details

**Table:**

Experimental. CrysAlisPro, Oxford Diffraction Ltd., Version 1.171.33.66 (release 28-04-2010 CrysAlis171 .NET) (compiled Apr 28 2010,14:27:37) Analytical numeric absorption correction using a multifaceted crystal model (Clark & Reid, 1995).
Geometry. All e.s.d.'s (except the e.s.d. in the dihedral angle between two l.s. planes) are estimated using the full covariance matrix. The cell e.s.d.'s are taken into account individually in the estimation of e.s.d.'s in distances, angles and torsion angles; correlations between e.s.d.'s in cell parameters are only used when they are defined by crystal symmetry. An approximate (isotropic) treatment of cell e.s.d.'s is used for estimating e.s.d.'s involving l.s. planes.
Refinement. Refinement of *F*^2^ against ALL reflections. The weighted *R*-factor *wR* and goodness of fit *S* are based on *F*^2^, conventional *R*-factors *R* are based on *F*, with *F* set to zero for negative *F*^2^. The threshold expression of *F*^2^ > σ(*F*^2^) is used only for calculating *R*-factors(gt) *etc*. and is not relevant to the choice of reflections for refinement. *R*-factors based on *F*^2^ are statistically about twice as large as those based on *F*, and *R*- factors based on ALL data will be even larger.

### Fractional atomic coordinates and isotropic or equivalent isotropic displacement parameters (Å^2^)

**Table:**

	*x*	*y*	*z*	*U*_iso_*/*U*_eq_	
O21	0.40317 (10)	−0.10768 (8)	0.21913 (9)	0.0790 (4)	
O22	0.44031 (11)	−0.17638 (7)	0.10322 (10)	0.0800 (5)	
H221	0.4499 (17)	−0.1704 (18)	0.023 (2)	0.120 (10)*	
O24	0.42558 (9)	−0.07834 (9)	−0.17086 (8)	0.0700 (4)	
O23	0.46000 (10)	−0.16051 (7)	−0.05594 (9)	0.0715 (4)	
N21	0.36865 (7)	0.02096 (7)	0.12139 (8)	0.0424 (3)	
C22	0.39606 (8)	−0.04057 (8)	0.07412 (10)	0.0401 (3)	
C23	0.40339 (8)	−0.03291 (8)	−0.02084 (10)	0.0404 (3)	
N24	0.38516 (7)	0.03671 (7)	−0.06538 (8)	0.0440 (3)	
C25	0.36067 (9)	0.09750 (8)	−0.01817 (11)	0.0459 (3)	
C26	0.35079 (9)	0.08917 (9)	0.07754 (11)	0.0447 (3)	
C27	0.41428 (11)	−0.11261 (10)	0.13755 (12)	0.0545 (4)	
C28	0.43074 (10)	−0.09444 (10)	−0.08791 (11)	0.0501 (4)	
C29	0.34350 (14)	0.17437 (11)	−0.07011 (14)	0.0694 (5)	
H291	0.3571	0.1692	−0.1326	0.104*	
H293	0.3746	0.2163	−0.0382	0.104*	
H292	0.2878	0.1868	−0.0727	0.104*	
C30	0.31919 (13)	0.15546 (10)	0.13190 (13)	0.0660 (5)	
H302	0.3141	0.1375	0.1942	0.099*	
H301	0.2679	0.1717	0.1014	0.099*	
H303	0.3553	0.1998	0.1349	0.099*	
O11	−0.09821 (8)	0.12180 (9)	0.68432 (9)	0.0681 (4)	
O12	−0.16977 (7)	0.06333 (9)	0.56697 (9)	0.0686 (4)	
H121	−0.1667 (15)	0.0483 (16)	0.4900 (19)	0.100 (8)*	
O13	−0.08820 (9)	0.05683 (9)	0.29824 (8)	0.0707 (4)	
O14	−0.16205 (7)	0.02872 (9)	0.40804 (9)	0.0681 (4)	
N11	0.02790 (7)	0.13911 (7)	0.59970 (8)	0.0420 (3)	
C12	−0.03649 (8)	0.10845 (8)	0.54690 (10)	0.0389 (3)	
C13	−0.03376 (8)	0.09184 (8)	0.45215 (10)	0.0401 (3)	
N14	0.03342 (8)	0.10634 (7)	0.41328 (8)	0.0444 (3)	
C15	0.09685 (9)	0.13481 (9)	0.46587 (11)	0.0464 (3)	
C16	0.09424 (9)	0.15177 (9)	0.56170 (11)	0.0454 (3)	
C17	−0.10577 (9)	0.09773 (9)	0.60402 (11)	0.0483 (4)	
C18	−0.09889 (10)	0.05765 (9)	0.38036 (12)	0.0504 (4)	
C19	0.17054 (12)	0.14820 (14)	0.41993 (14)	0.0704 (5)	
H191	0.1617	0.1297	0.3563	0.106*	
H193	0.2143	0.1194	0.4534	0.106*	
H192	0.1829	0.2040	0.4207	0.106*	
C20	0.16447 (11)	0.18443 (13)	0.62319 (13)	0.0666 (5)	
H203	0.1512	0.1909	0.6857	0.100*	
H201	0.1789	0.2351	0.5994	0.100*	
H202	0.2086	0.1483	0.6240	0.100*	
O15	0.0000	0.27959 (11)	0.7500	0.0753 (6)	
O25	0.25338 (8)	−0.01032 (11)	0.27697 (12)	0.0805 (5)	
Li11	0.0000	0.1656 (2)	0.7500	0.0537 (9)	
Li21	0.36552 (17)	−0.01282 (17)	0.2742 (2)	0.0548 (6)	
Li12	0.0000	0.1074 (2)	0.2500	0.0558 (9)	
O16	0.0000	0.21954 (12)	0.2500	0.1067 (11)	
H161	0.0175 (17)	0.2467 (17)	0.2957 (18)	0.105 (9)*	
H151	0.016 (2)	0.310 (2)	0.709 (2)	0.160 (15)*	
H251	0.2275 (17)	−0.0304 (16)	0.323 (2)	0.105 (9)*	
H252	0.220 (2)	−0.0018 (19)	0.233 (2)	0.120 (11)*	

### Atomic displacement parameters (Å^2^)

**Table:**

	*U*^11^	*U*^22^	*U*^33^	*U*^12^	*U*^13^	*U*^23^
O21	0.1200 (12)	0.0716 (8)	0.0510 (7)	0.0362 (8)	0.0324 (8)	0.0182 (6)
O22	0.1352 (13)	0.0487 (6)	0.0592 (8)	0.0289 (7)	0.0239 (8)	0.0074 (6)
O24	0.0872 (9)	0.0831 (8)	0.0404 (7)	0.0274 (7)	0.0114 (6)	−0.0068 (6)
O23	0.1044 (11)	0.0566 (7)	0.0541 (7)	0.0250 (7)	0.0130 (7)	−0.0077 (6)
N21	0.0435 (6)	0.0453 (6)	0.0380 (6)	0.0021 (5)	0.0043 (5)	−0.0027 (5)
C22	0.0396 (7)	0.0425 (7)	0.0381 (7)	0.0009 (6)	0.0046 (6)	−0.0022 (6)
C23	0.0366 (7)	0.0443 (7)	0.0397 (8)	−0.0007 (6)	0.0028 (6)	−0.0025 (6)
N24	0.0439 (6)	0.0491 (7)	0.0391 (7)	0.0010 (5)	0.0053 (5)	0.0019 (5)
C25	0.0455 (8)	0.0444 (7)	0.0474 (9)	0.0011 (6)	0.0041 (7)	0.0015 (6)
C26	0.0451 (8)	0.0436 (7)	0.0447 (8)	0.0015 (6)	0.0030 (6)	−0.0043 (6)
C27	0.0681 (10)	0.0500 (8)	0.0465 (9)	0.0109 (7)	0.0124 (8)	0.0059 (7)
C28	0.0506 (8)	0.0575 (9)	0.0420 (9)	0.0052 (7)	0.0057 (7)	−0.0085 (7)
C29	0.0900 (14)	0.0540 (9)	0.0656 (12)	0.0134 (9)	0.0150 (10)	0.0126 (8)
C30	0.0877 (13)	0.0502 (9)	0.0603 (11)	0.0150 (9)	0.0105 (10)	−0.0088 (8)
O11	0.0600 (7)	0.0964 (9)	0.0515 (7)	−0.0178 (7)	0.0216 (6)	−0.0156 (7)
O12	0.0442 (6)	0.1023 (10)	0.0612 (8)	−0.0187 (6)	0.0142 (6)	−0.0090 (7)
O13	0.0803 (9)	0.0879 (9)	0.0423 (7)	−0.0275 (7)	0.0018 (6)	−0.0057 (6)
O14	0.0529 (7)	0.0927 (9)	0.0578 (7)	−0.0220 (6)	0.0031 (6)	−0.0094 (7)
N11	0.0428 (6)	0.0435 (6)	0.0399 (6)	−0.0034 (5)	0.0060 (5)	0.0022 (5)
C12	0.0402 (7)	0.0363 (6)	0.0405 (8)	0.0005 (5)	0.0055 (6)	0.0034 (5)
C13	0.0427 (7)	0.0367 (6)	0.0407 (8)	0.0013 (5)	0.0047 (6)	0.0029 (6)
N14	0.0493 (7)	0.0442 (6)	0.0411 (7)	−0.0022 (5)	0.0107 (6)	0.0021 (5)
C15	0.0447 (8)	0.0474 (8)	0.0487 (8)	−0.0029 (6)	0.0115 (7)	0.0027 (6)
C16	0.0437 (8)	0.0467 (7)	0.0459 (8)	−0.0045 (6)	0.0068 (7)	0.0050 (6)
C17	0.0431 (8)	0.0536 (8)	0.0494 (9)	−0.0010 (6)	0.0102 (7)	0.0003 (7)
C18	0.0527 (9)	0.0500 (8)	0.0474 (9)	−0.0041 (7)	0.0019 (7)	0.0003 (7)
C19	0.0571 (11)	0.0933 (14)	0.0649 (12)	−0.0168 (10)	0.0236 (9)	−0.0071 (10)
C20	0.0524 (10)	0.0911 (13)	0.0556 (10)	−0.0229 (9)	0.0045 (8)	−0.0006 (9)
O15	0.0985 (15)	0.0525 (9)	0.0844 (14)	0.000	0.0487 (12)	0.000
O25	0.0477 (7)	0.1254 (13)	0.0678 (9)	0.0029 (7)	0.0043 (7)	0.0356 (9)
Li11	0.058 (2)	0.056 (2)	0.048 (2)	0.000	0.0099 (18)	0.000
Li21	0.0579 (15)	0.0626 (15)	0.0455 (14)	0.0016 (13)	0.0125 (12)	−0.0005 (12)
Li12	0.071 (2)	0.051 (2)	0.046 (2)	0.000	0.0093 (19)	0.000
O16	0.194 (3)	0.0423 (9)	0.0687 (14)	0.000	−0.0406 (17)	0.000

### Geometric parameters (Å, º)

**Table:**

O21—C27	1.214 (2)	C27—Li21	2.786 (3)
Li11—O15	1.915 (4)	C29—H291	0.9600
Li11—O11	1.9473 (19)	C29—H293	0.9600
Li11—O11^i^	1.9472 (19)	C29—H292	0.9600
Li11—N11^i^	2.3128 (14)	C30—H302	0.9600
Li11—N11	2.3129 (14)	C30—H301	0.9600
Li12—O16	1.884 (4)	C30—H303	0.9600
Li12—O13	1.920 (2)	O11—C17	1.215 (2)
Li12—O13^ii^	1.920 (2)	O12—C17	1.282 (2)
Li12—N14^ii^	2.3446 (12)	O12—H121	1.14 (3)
Li12—N14	2.3446 (12)	O13—C18	1.217 (2)
Li21—O25	1.902 (3)	O14—C18	1.282 (2)
Li21—O21	1.923 (3)	O14—H121	1.24 (3)
Li21—O24^iii^	1.949 (3)	N11—C16	1.3255 (19)
Li21—N21	2.276 (3)	N11—C12	1.3481 (19)
Li21—N24^iii^	2.324 (3)	C12—C13	1.397 (2)
O22—C27	1.281 (2)	C12—C17	1.524 (2)
O22—H221	1.18 (3)	C13—N14	1.3499 (19)
O24—C28	1.215 (2)	C13—C18	1.523 (2)
O24—Li21^iv^	1.949 (3)	N14—C15	1.321 (2)
O23—C28	1.277 (2)	C15—C16	1.413 (2)
O23—H221	1.19 (3)	C15—C19	1.499 (2)
N21—C26	1.3242 (19)	C16—C20	1.492 (2)
N21—C22	1.3509 (18)	C19—H191	0.9600
C22—C23	1.393 (2)	C19—H193	0.9600
C22—C27	1.523 (2)	C19—H192	0.9600
C23—N24	1.3502 (19)	C20—H203	0.9600
C23—C28	1.525 (2)	C20—H201	0.9600
N24—C25	1.3214 (19)	C20—H202	0.9600
N24—Li21^iv^	2.324 (3)	O15—H151	0.85 (3)
C25—C26	1.414 (2)	O25—H251	0.91 (3)
C25—C29	1.502 (2)	O25—H252	0.80 (3)
C26—C30	1.497 (2)	O16—H161	0.82 (3)
			
C27—O21—Li21	123.64 (14)	N11—C16—C15	120.13 (14)
C27—O22—H221	113.5 (14)	N11—C16—C20	117.77 (14)
C28—O24—Li21^iv^	122.81 (14)	C15—C16—C20	122.09 (14)
C28—O23—H221	111.8 (14)	O11—C17—O12	121.80 (14)
C26—N21—C22	119.64 (13)	O11—C17—C12	118.52 (14)
C26—N21—Li21	130.06 (12)	O12—C17—C12	119.67 (14)
C22—N21—Li21	110.26 (12)	O13—C18—O14	121.93 (15)
N21—C22—C23	120.10 (13)	O13—C18—C13	118.59 (14)
N21—C22—C27	111.27 (12)	O14—C18—C13	119.45 (14)
C23—C22—C27	128.63 (13)	C15—C19—H191	109.5
N24—C23—C22	120.12 (13)	C15—C19—H193	109.5
N24—C23—C28	110.88 (13)	H191—C19—H193	109.5
C22—C23—C28	129.00 (13)	C15—C19—H192	109.5
C25—N24—C23	119.55 (13)	H191—C19—H192	109.5
C25—N24—Li21^iv^	128.96 (12)	H193—C19—H192	109.5
C23—N24—Li21^iv^	108.47 (12)	C16—C20—H203	109.5
N24—C25—C26	120.45 (13)	C16—C20—H201	109.5
N24—C25—C29	117.55 (14)	H203—C20—H201	109.5
C26—C25—C29	122.00 (14)	C16—C20—H202	109.5
N21—C26—C25	120.07 (13)	H203—C20—H202	109.5
N21—C26—C30	118.02 (14)	H201—C20—H202	109.5
C25—C26—C30	121.90 (14)	Li11—O15—H151	127 (2)
O21—C27—O22	122.16 (15)	Li21—O25—H251	125.5 (18)
O21—C27—C22	118.70 (14)	Li21—O25—H252	126 (2)
O22—C27—C22	119.14 (14)	H251—O25—H252	107 (3)
O21—C27—Li21	35.08 (10)	O15—Li11—O11^i^	112.18 (11)
O22—C27—Li21	157.24 (13)	O15—Li11—O11	112.18 (11)
C22—C27—Li21	83.62 (10)	O11^i^—Li11—O11	135.6 (2)
O24—C28—O23	121.55 (15)	O15—Li11—N11^i^	101.08 (10)
O24—C28—C23	118.69 (14)	O11^i^—Li11—N11^i^	74.72 (6)
O23—C28—C23	119.74 (14)	O11—Li11—N11^i^	96.81 (8)
C25—C29—H291	109.5	O15—Li11—N11	101.08 (10)
C25—C29—H293	109.5	O11^i^—Li11—N11	96.80 (8)
H291—C29—H293	109.5	O11—Li11—N11	74.72 (6)
C25—C29—H292	109.5	N11^i^—Li11—N11	157.8 (2)
H291—C29—H292	109.5	O25—Li21—O21	114.19 (17)
H293—C29—H292	109.5	O25—Li21—O24^iii^	116.34 (16)
C26—C30—H302	109.5	O21—Li21—O24^iii^	129.46 (17)
C26—C30—H301	109.5	O25—Li21—N21	99.20 (13)
H302—C30—H301	109.5	O21—Li21—N21	76.11 (10)
C26—C30—H303	109.5	O24^iii^—Li21—N21	96.95 (13)
H302—C30—H303	109.5	O25—Li21—N24^iii^	90.02 (12)
H301—C30—H303	109.5	O21—Li21—N24^iii^	104.63 (13)
C17—O11—Li11	124.56 (11)	O24^iii^—Li21—N24^iii^	74.42 (11)
C17—O12—H121	111.3 (13)	N21—Li21—N24^iii^	169.57 (15)
C18—O13—Li12	124.25 (12)	O25—Li21—C27	114.39 (14)
C18—O14—H121	110.6 (12)	O21—Li21—C27	21.28 (6)
C16—N11—C12	119.61 (13)	O24^iii^—Li21—C27	124.99 (14)
C16—N11—Li11	129.62 (11)	N21—Li21—C27	54.84 (7)
C12—N11—Li11	110.74 (10)	N24^iii^—Li21—C27	125.35 (12)
N11—C12—C13	120.10 (12)	O16—Li12—O13	116.26 (11)
N11—C12—C17	111.24 (12)	O16—Li12—O13^ii^	116.26 (11)
C13—C12—C17	128.65 (13)	O13—Li12—O13^ii^	127.5 (2)
N14—C13—C12	120.07 (13)	O16—Li12—N14^ii^	90.44 (10)
N14—C13—C18	111.17 (13)	O13—Li12—N14^ii^	104.99 (7)
C12—C13—C18	128.75 (13)	O13^ii^—Li12—N14^ii^	74.61 (5)
C15—N14—C13	119.52 (13)	O16—Li12—N14	90.44 (10)
C15—N14—Li12	130.23 (11)	O13—Li12—N14	74.61 (5)
C13—N14—Li12	107.90 (10)	O13^ii^—Li12—N14	104.99 (7)
N14—C15—C16	120.53 (14)	N14^ii^—Li12—N14	179.1 (2)
N14—C15—C19	117.49 (15)	Li12—O16—H161	123.6 (19)
C16—C15—C19	121.98 (15)		

Symmetry codes: (i) −*x*, *y*, −*z*+3/2; (ii) −*x*, *y*, −*z*+1/2; (iii) *x*, −*y*, *z*+1/2; (iv) *x*, −*y*, *z*−1/2.

### Hydrogen-bond geometry (Å, º)

**Table:**

*D*—H···*A*	*D*—H	H···*A*	*D*···*A*	*D*—H···*A*
O22—H221···O23	1.18 (3)	1.19 (3)	2.3693 (18)	177 (3)
O12—H121···O14	1.14 (3)	1.24 (3)	2.3777 (18)	177 (3)
O16—H161···O22^v^	0.82 (3)	2.01 (3)	2.8268 (18)	173 (3)
O15—H151···O24^v^	0.85 (3)	2.22 (4)	2.989 (2)	150 (3)
O15—H151···O23^v^	0.85 (3)	2.34 (3)	3.1201 (14)	153 (3)
O25—H251···O12^vi^	0.91 (3)	2.03 (3)	2.938 (2)	174 (2)
O25—H252···O14^ii^	0.80 (3)	2.20 (3)	2.975 (2)	163 (3)
O25—H252···O13^ii^	0.80 (3)	2.43 (3)	3.078 (2)	139 (3)

Symmetry codes: (ii) −*x*, *y*, −*z*+1/2; (v) −*x*+1/2, *y*+1/2, −*z*+1/2; (vi) −*x*, −*y*, −*z*+1.
